# Triglycerides and ovarian response in diminished ovarian reserve: a two-sample mendelian randomization and clinical mediation study

**DOI:** 10.3389/fendo.2026.1914533

**Published:** 2026-09-15

**Authors:** Xiaoju Wan, Min Yu, Xingwu Wu, Zhihui Huang, Jun Tan

**Affiliations:** Reproductive Medicine Center, Jiangxi Maternal and Child Health Hospital, Nanchang, China

**Keywords:** assisted reproductive technology, diminished ovarian reserve, lipid metabolism, mediation analysis, Mendelian randomization, triglycerides

## Abstract

**Background:**

Diminished ovarian reserve (DOR) impairs assisted reproduction. Whether triglyceride (TG) plays a causal role in ovarian reserve remains unclear.

**Methods:**

We performed a bidirectional two-sample Mendelian randomization (MR) analysis using female-specific GWAS data to assess causality between metabolic traits and ovarian reserve markers (AMH, FSH). Findings were validated in a retrospective cohort of 8,851 DOR women undergoing their first IVF/ICSI cycle, using mediation analysis to elucidate pathways to oocyte yield.

**Results:**

MR revealed that genetically predicted TG was causally associated with lower FSH (IVW β=-0.096, FDR = 5.28×10^-5^), with no evidence of reverse causation. Clinical validation confirmed this association. Mediation analysis uncovered that TG exerted a direct negative effect on oocyte yield, but concurrently lowered FSH, creating a suppression effect that partially counteracted this detriment (indirect effect: β = 0.0073, 95% CI: 0.0029 to 0.0117, p = 0.0021; suppression proportion: 23.6%). TG did not show a causal effect on AMH in MR (IVW OR = 1.035, 95% CI: 0.965-1.111, p = 0.338), and clinical mediation through AMH was not robust. Despite impacting oocyte yield, TG did not independently predict clinical pregnancy or live birth; age and AMH remained dominant.

**Conclusion:**

This study provides genetic evidence for a causal role of TG in lowering FSH and identifies a suppression pathway through which TG affects oocyte yield. These findings provide additional insight into the relationship between lipid metabolism and ovarian reserve, and highlight TG as a potential modifiable factor worthy of further investigation.

## 1 Introduction

Diminished ovarian reserve (DOR) is a prevalent clinical condition characterized by a reduction in the quantity and/or quality of oocytes, leading to declined ovarian function and female subfertility (1). It is typically defined by biochemical markers, including low serum anti-Müllerian hormone (AMH) levels and elevated basal follicle-stimulating hormone (FSH) (2). DOR poses a significant challenge in assisted reproductive technology (ART), as it is strongly associated with poor ovarian response, reduced oocyte yield, and consequently, lower live birth rates (3, 4). The current clinical assessment of ovarian reserve primarily relies on age, AMH, and FSH, which, while useful, offer limited insight into the underlying pathophysiological drivers of ovarian aging (5).

Accumulating evidence from both animal models and observational clinical studies has suggested a link between metabolic metabolic and ovarian function (6–8). Conditions such as elevated body mass index (BMI), type 2 diabetes (T2D), and metabolic syndrome have been correlated with altered levels of AMH, fewer retrieved oocytes, as well as poorer ART outcome (9–12). However, critical questions remain unresolved due to inherent limitations of these studies. First, the direction of causality is unclear: it is unknown whether metabolic dysregulation is a driver of the pathogenesis of DOR or merely a consequence of the endocrine changes associated with ovarian aging itself, as conventional observational studies are inherently confounded by reverse causality and potential confounding factors. Second, while animal studies imply potential mechanisms, the specific clinical pathways through which metabolic factors, particularly blood lipids, influence ovarian response and IVF outcomes in infertile women remain largely unexplored and unquantified.

Mendelian randomization (MR), an epidemiological method that uses genetic variants as instrumental variables, provides a powerful approach to infer causality by minimizing confounding (13, 14). Applying MR to female-specific genetic data can help disentangle the direction of causality between metabolic traits and ovarian function. Nevertheless, establishing causality is only the first step; understanding the mediating pathways is essential for clinical translation.

To address these gaps, we hypothesized that triglycerides (TG) exert a causal effect on ovarian reserve and that this effect mediates key ART outcomes. We conducted an integrated two-stage study. First, we performed a two-sample MR analysis to assess the causal links between genetically predicted metabolic indices and ovarian reserve markers. Second, we rigorously validated these findings in a large, well-characterized clinical cohort of DOR patients. A key innovation of our clinical analysis was the use of causal mediation models to quantify the extent to which TG influence oocyte yield through FSH. By integrating genetic causality with clinical mediation analysis, this study aims to elucidate the role of TG in ovarian reserve and identify potential targets for improving the management of DOR.

## 2 Materials and methods

### 2.1 Study design

This study employed an integrated two-stage design to investigate the role of metabolic factors in DOR (1): a two-sample MR analysis to assess causal relationships between metabolic traits and ovarian reserve markers; and (2) a retrospective clinical cohort study of 8,851 DOR patients undergoing their first IVF/ICSI cycle to validate and further explore the MR findings. The study was approved by the Reproductive Medicine Ethics Committee of Jiangxi Maternal and Child Health Hospital (Approval No. SZYY-202509). Informed consent was waived due to the retrospective design. Reporting follows STROBE-MR guidelines (**Supplementary Table 1**).

### 2.2 Part I: Mendelian randomization study

#### 2.2.1 Data sources and GWAS summary statistics

Female-specific genome-wide association study (GWAS) summary statistics for metabolic traits (body mass index [BMI], waist-to-hip ratio [WHR]), lipid traits (triglycerides [TG], low-density [LDL-c] and high-density lipoprotein cholesterol [HDL-c]), glycemic traits (type 2 diabetes [T2D], fasting insulin, fasting glucose), and ovarian reserve markers (anti-Müllerian hormone [AMH], follicle-stimulating hormone [FSH]) were obtained from publicly available consortia (15, 16). **Supplementary Table 2** provides detailed data sources and cohort descriptions.

#### 2.2.2 Genetic instrument selection

Single nucleotide polymorphisms (SNPs) robustly associated (p < 5 × 10^-8^) with each metabolic trait were selected as instrumental variables. These SNPs were clumped (linkage disequilibrium r² < 0.001, distance = 10,000 kb) to ensure independence. Palindromic SNPs were removed during data harmonization. The strength of each genetic instrument was assessed using the F-statistic, with F > 10 indicating a strong instrument and minimizing weak instrument bias.

#### 2.2.3 Statistical analysis for MR

The inverse-variance weighted (IVW) method served as the primary analysis to estimate causal effects. To validate the robustness of these findings and assess potential violations of MR assumptions, we conducted a suite of sensitivity analyses. These included MR-Egger regression to test for directional horizontal pleiotropy; the weighted median method to provide a consistent estimate under the assumption that at least 50% of the instrumental variables are valid; Cochran’s Q statistic to quantify heterogeneity. For associations with significant heterogeneity, MR-PRESSO and leave-one-out analyses were additionally performed to detect outlier SNPs and to ensure that no single SNP was driving the causal estimate. All analyses were performed using the “TwoSampleMR” and “MR-PRESSO” packages in R, with a false discovery rate (FDR) < 0.05 considered significant for multiple testing correction.

### 2.3 Part II: clinical cohort study

#### 2.3.1 Study population, design, and data collection

A retrospective cohort was established from patients undergoing their first *in vitro* fertilization/intracytoplasmic sperm injection (IVF/ICSI) cycle at our center between January 2010 and October 2025. The inclusion criteria were: 1) infertility duration ≥ 1 year; 2) a diagnosis of DOR, defined as AMH < 1.1 ng/ml, antral follicle count (AFC) < 7, or basal FSH ≥ 10 IU/L. Key exclusion criteria included chromosomal abnormalities, polycystic ovary syndrome (PCOS), diabetes mellitus, congenital uterine malformations, severe hydrosalpinx, history of recurrent miscarriage, obstructive/non-obstructive azoospermia, and donor sperm/donor oocyte cycle.

For all enrolled patients, we collected the following baseline characteristics: female age, body mass index (BMI), infertility type and duration, ovarian reserve markers (basal FSH, luteinizing hormone [LH], estradiol [E_2_], AMH, and AFC), and fasting serum lipid profiles (triglycerides [TG], total cholesterol [TC], high-density lipoprotein cholesterol [HDL-c], and low-density lipoprotein cholesterol [LDL-c]).

#### 2.3.2 Treatment protocols and clinical procedures

The ovarian stimulation strategy was individualized based on the patient’s age, BMI, and ovarian reserve. The treatment protocols included Gonadotropin-Releasing Hormone (GnRH) agonist protocols (long, ultralong, or short), GnRH antagonist protocols, and other protocols (such as mild stimulation or Progestin-Primed Ovarian Stimulation, PPOS). The decision to perform fresh embryo transfer or adopt a “freeze-all” strategy was made by the physician based on the patient’s ovarian response and overall clinical condition.

In fresh embryo transfer cycles, luteal phase support was routinely administered using dydrogesterone combined with a progesterone sustained-release vaginal gel. Biochemical pregnancy was defined by a positive serum human chorionic gonadotropin (hCG) test 14 days after embryo transfer. Clinical pregnancy was confirmed five weeks later via ultrasonographic visualization of a gestational sac with primitive cardiac activity. Follow-up was conducted to record birth outcomes.

#### 2.3.3 Outcome measures

The primary outcome was the number of oocytes retrieved. Secondary outcomes, which were analyzed only for the subset of patients undergoing fresh embryo transfer, included the biochemical pregnancy rate (defined as the number of cycles with positive hCG per transfer cycle × 100%), clinical pregnancy rate (number of cycles with ultrasonographic confirmation of a gestational sac per transfer cycle × 100%), miscarriage rate (number of spontaneous or therapeutic abortions per clinical pregnancy cycle × 100%), and live birth rate (number of deliveries with a live infant at ≥28 weeks of gestation per transfer cycle × 100%). Additionally, we collected key process parameters, including total gonadotropin (Gn) dose and duration, serum hormone levels on the day of hCG trigger, normal fertilization rate, and high-quality embryo rate.

#### 2.3.4 Statistical analysis

Statistical analyses were performed using R software. Continuous variables are summarized as mean ± standard deviation or median [IQR] based on their normality, assessed by the Shapiro-Wilk test, and were compared using ANOVA or the Kruskal-Wallis test, as appropriate. Categorical variables are presented as n (%) and were compared using the Chi-square or Fisher’s exact test. Correlations between metabolic factors and ovarian reserve markers were assessed using Pearson or Spearman correlation coefficients. Multivariable linear and logistic regression models were employed to identify independent predictors of hormone levels and clinical outcomes, respectively, adjusting for relevant confounders such as age and treatment protocol. For the primary outcome (oocyte yield), we constructed sequential multivariable linear regression models to assess the independent contribution of metabolic factors after adjusting for known confounders (see **Supplementary Table 11** for model specifications). Model 4 (adjusted for age, infertility factors, and ovarian reserve markers) was designated as the primary model, as it adjusts for all baseline confounders without conditioning on treatment-related variables. Models 5–6 are presented as sensitivity analyses. To elucidate the pathways through which metabolic factors influence oocyte yield, we conducted mediation analyses using both a traditional causal steps approach with bootstrapping (1000 resamples) and a Bayesian framework (Variational Bayes method, VB-RobM) to ensure robust and probabilistic inference. A two-tailed p-value < 0.05 was considered statistically significant.

## 3 Results

### 3.1 Part I: MR analyses reveal causal links from metabolic traits to ovarian reserve

After FDR correction (FDR < 0.05), the most robust causal association identified was between genetically predicted TG and lower FSH levels (IVW β = -0.096, 95% CI: -0.136 to -0.056, FDR = 5.28×10^-5^) (**Figure 1**). This finding was consistently supported across all supplementary MR methods (all p < 0.01). Sensitivity analyses confirmed the robustness of this association; no directional pleiotropy was detected (MR-Egger intercept p = 0.564), though significant heterogeneity was present (Cochran’s Q p < 0.001). Other metabolic traits showed less consistent associations with FSH, and no metabolic trait demonstrated a robust causal effect on AMH (all FDR > 0.05; see **Supplementary Table 4** for complete results).

**Figure 1:**
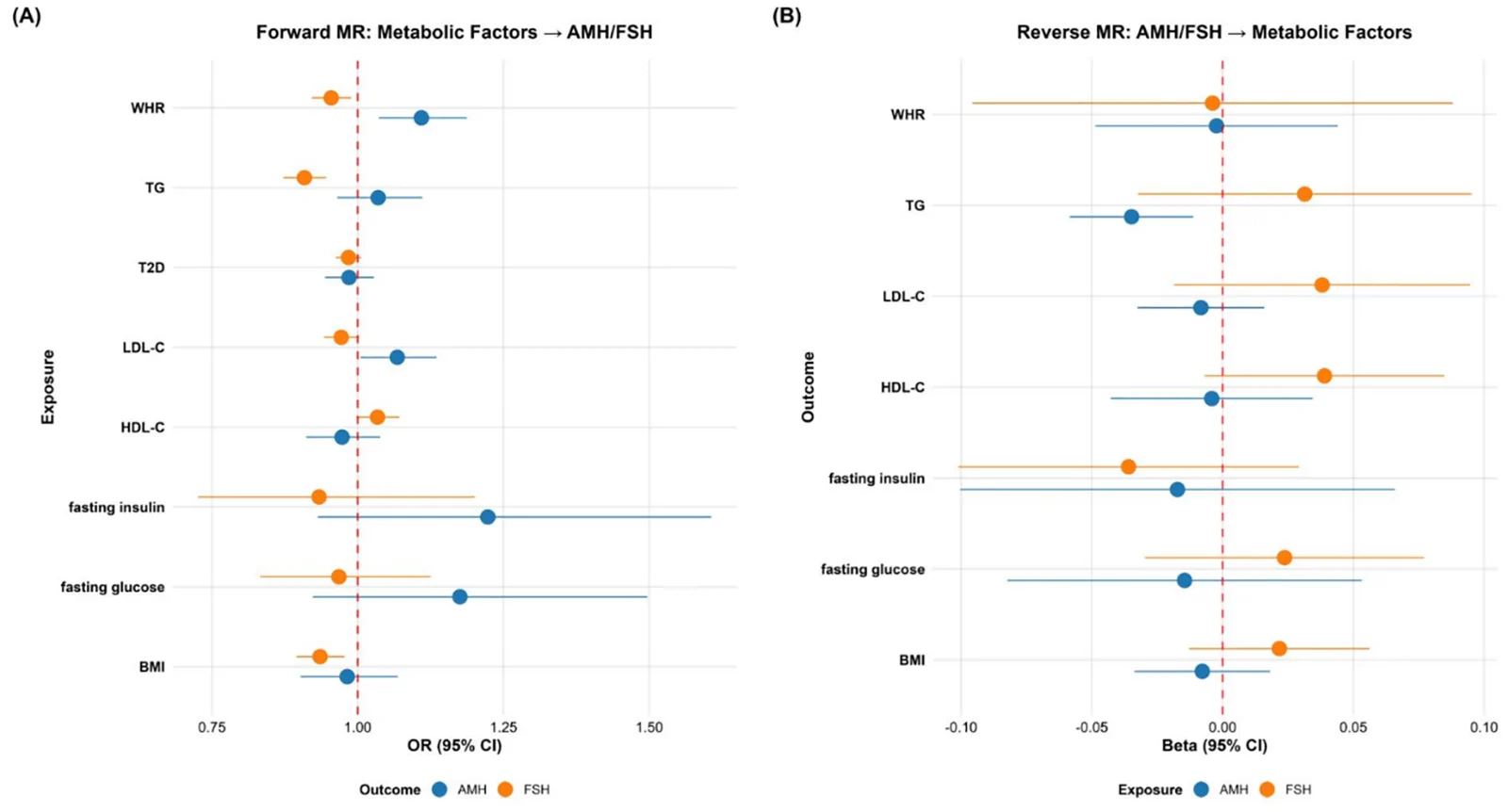
Bidirectional mendelian randomization analysis of metabolic factors and ovarian reserve biomarkers. **(A)** Causal effects of genetically predicted metabolic factors on ovarian reserve markers (AMH and FSH). **(B)** Reverse analysis assessing the causal effects of genetically predicted ovarian reserve markers on metabolic factors. Effect estimates and 95% confidence intervals are derived from the inverse-variance weighted method. The dashed red line indicates the null effect.

In reverse MR analyses, no significant associations were identified after FDR correction (all FDR > 0.05). While AMH→TG showed a nominally significant association (β = -0.035, p = 0.004), this did not survive multiple testing correction (FDR = 0.052). Further details, including data sources (**Supplementary Table 2**), genetic instruments (**Supplementary Table 3**), complete MR results (**Supplementary Tables 4**, **S6**), and sensitivity analyses (**Supplementary Tables 5**, **S7**), are available in the **Supplementary Material**.

### 3.2 Part II: clinical cohort study validates and elucidates causal pathways

Baseline characteristics of this cohort are summarized in **Supplementary Table 8A**.

#### 3.2.1 Metabolic profiles correlate with ovarian reserve markers

Bivariate correlation analysis revealed a significant negative correlation between TG and FSH (r = -0.055, p < 0.001), consistent with the MR finding (**Supplementary Table 9**). In multivariable linear regression models adjusting for age and LDL-c, TG levels exhibited a significant inverse association with FSH (β = -0.044, p = 0.001) (**Table 1**). BMI similarly demonstrated a robust negative association with FSH (β = -0.416, p < 0.001). Sensitivity analyses incorporating comprehensive lipid profiles confirmed the specific association of TG with FSH (β = -0.059, p < 0.001; **Supplementary Table 10**).

**Table 1:** Multivariable linear regression analysis of metabolic factors and ovarian reserve markers in women with DOR undergoing their first IVF/ICSI cycle.

Predictor	Log (FSH)		Log (AMH)	
	β (SE)	P-value	β (SE)	P-value
Log (BMI)	-0.416 (0.045)	<0.001	-0.405 (0.063)	<0.001
Log (TG)	-0.044 (0.013)	0.001	-0.034 (0.018)	0.065
Log (LDL-c)	0.025 (0.020)	0.223	0.029 (0.028)	0.299
Age	0.006 (0.001)	<0.001	-0.019 (0.002)	<0.001
Model statistics				
N	6,048		6,576	
R²	0.024		0.035	

Results are from multivariable linear regression models, with log(FSH) or log(AMH) as the outcome, adjusting for all other variables presented in the table. All continuous predictors and outcomes were log-transformed to approximate normal distributions. β, regression coefficient; SE, standard error. The R² value represents the proportion of variance in the outcome explained by the model. The sample size (N) reflects complete-case analysis after excluding missing values in any of the included variables; the full cohort was 8,851.

#### 3.2.2 Impact of metabolic factors on oocyte yield

In sequential multivariable linear regression models (**Supplementary Table 11**), TG showed a significant negative association with oocyte yield in the primary model (Model 4: β = -0.323, p<0.01), adjusted for all baseline confounders including age, infertility factors, and ovarian reserve markers (FSH, AMH, AFC) (**Table 2**). This association remained consistent in the fully adjusted model (Model 6: β = -0.235, p<0.05), which additionally adjusted for treatment-related variables (**Supplementary Table 11**). As expected, AMH was the strongest predictor of oocyte yield (β = 2.157, p < 0.001).

**Table 2:** Multivariable linear regression analysis of determinants for oocyte yield in women with DOR (primary model).

Predictor	β (SE)
Metabolic factors	
Log (BMI)	-0.586 (0.380)
Log (TG)	-0.323 (0.120) **
Log (TC)	0.237 (0.545)
Log (HDL-c)	-0.310 (0.270)
Log (LDL-c)	-0.228 (0.290)
Ovarian reserve	
Log (FSH)	-0.473 (0.109) ***
Log (AMH)	2.157 (0.066) ***
Log (AFC)	0.847 (0.088) ***
Demographics	
Age	-0.141 (0.010) ***
Primary infertility	-0.087 (0.110)
Infertility duration	-0.004 (0.013)
Model statistics	
N	5,187
R²	0.310

Model 4 is the primary model, adjusted for all baseline confounders (age, infertility factors, and ovarian reserve markers including AMH, FSH, and AFC) without conditioning on treatment-related variables. All continuous variables were log-transformed. *P < 0.05, **P < 0.01, ***P < 0.001. The R² of 0.310 indicates that the model explains approximately 31% of the variance in oocyte yield; the remaining variance reflects unmeasured factors inherent to ovarian response. The sample size (N = 5,187) reflects complete-case analysis after excluding missing values in any of the included variables; the full cohort was 8,851. Models 5–6, which additionally adjust for treatment-related variables, are provided in **Supplementary Table 11** as sensitivity analyses.

#### 3.3.3 FSH mediate the effect of triglycerides on oocyte yield

Restricted cubic spline regression revealed no significant evidence of non-linearity for the association between TG and FSH (p = 0.252), supporting the use of linear regression models (**Supplementary Table 8D**). Mediation analysis revealed a suppression effect in the TG-FSH pathway (**Figure 2A**). While TG exerted a direct negative effect on oocyte yield (direct effect: β = -0.042, p = 0.0008), it simultaneously lowered FSH, which partially counteracted this negative impact. The indirect effect through FSH was positive (β = 0.0073, 95% CI: 0.0029 to 0.0117, p = 0.0021), representing a suppression proportion of 23.6% (**Supplementary Table 12**). Bayesian mediation analysis confirmed this finding (**Figure 2B**, **Supplementary Table 13**).

**Figure 2:**
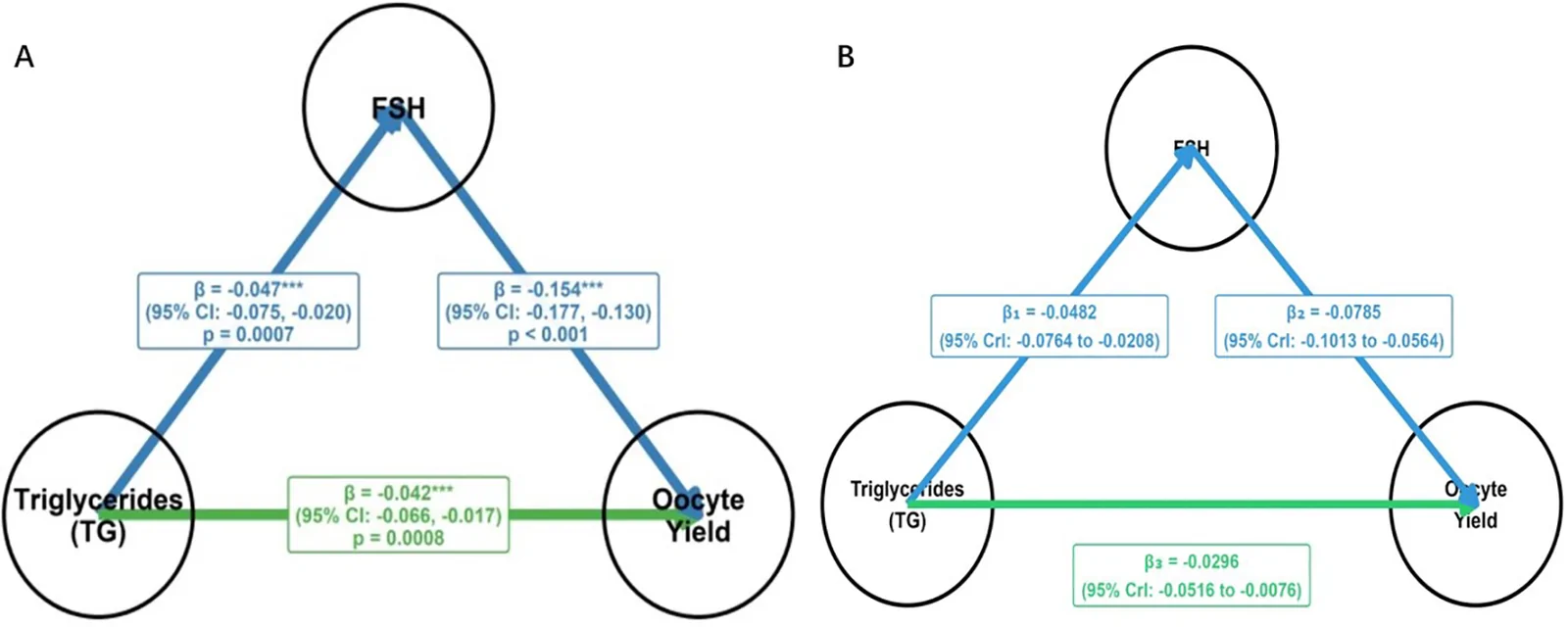
Mediation analysis of the TG-FSH-oocyte yield. **(A)** Traditional frequentist mediation analysis. TG exerts a direct negative effect on oocyte yield (c' = -0.042, p = 0.0008) while concurrently lowering FSH (a = -0.047, p < 0.001); lower FSH is associated with higher oocyte yield (b = -0.154, p < 0.001). The indirect effect through FSH is positive (0.0073, 95% CI: 0.0029–0.0117, p = 0.0021), indicating a suppression effect (suppression proportion: 23.6%). **(B)** Bayesian mediation analysis. Posterior distributions confirm the suppression effect, with indirect effect = 0.0038 (95% CrI: 0.0015–0.0065) and P (b ≠ 0) = 1.00. Both analyses adjusted for age, BMI, infertility type, duration, and AFC. Minor numerical differences between methods reflect their distinct statistical frameworks; both converge on the same qualitative conclusion. CrI, credible interval. See **Supplementary Table 12, 13** for full results. *P < 0.05, **P < 0.01, ***P < 0.001.

We also explored AMH as a potential mediator in exploratory analyses; however, these findings were not supported by MR (TG→AMH: OR = 1.035, p = 0.338) and are therefore not pursued as a primary conclusion (see **Supplementary Tables 12**, **13**).

#### 3.3.4 AMH, but not metabolic traits, predicts ultimate ART success

In the 4,022 cycles with fresh embryo transfers, clinical pregnancy rate was 55.8% (2,246/4,022), live birth rate was 44.2% (1,734/3,920), and miscarriage rate was 19.1% (410/2,144). Multivariable logistic regression revealed that AMH was significantly associated with both clinical pregnancy (aOR 1.12, 95% CI 1.03-1.22; p = 0.010) and live birth (aOR 1.13, 95% CI 1.03-1.23; p = 0.007). In contrast, neither TG nor BMI showed significant associations with any clinical outcome (**Table 3**). Age remained the strongest predictor across all outcomes.

**Table 3:** Multivariable logistic regression analysis of factors associated with clinical outcomes in fresh embryo transfer cycles (N = 4,022).

Variable	Category/(unit)	Clinical pregnancy	Miscarriage	Live birth
		aOR (95% CI)	aOR (95% CI)	aOR (95% CI)
Patient characteristics				
Age	(per 1 SD)	0.70(0.63-0.76) ***	1.45(1.23-1.70) ***	0.66 (0.60-0.72) ***
Primary infertility	Yes vs. No	0.77(0.64-0.93) **	0.97(0.69-1.34)	0.81 (0.67-0.97) *
Infertility duration	(per 1 SD)	0.92(0.84-1.00) *	0.97(0.84-1.11)	0.95 (0.87-1.04)
Treatment factors				
fertilization method	ICSI vs. IVF	0.77(0.63-0.94) *	1.09(0.76-1.56)	0.76 (0.62-0.93) **
Embryo Stage	Blastocyst vs. Cleavage	2.34(1.73-3.16) ***	0.54(0.31-0.94) *	2.10 (1.53-2.87) ***
Embryo transfer	Multiple vs. Single	2.04(1.62-2.56) ***	0.70(0.46-1.07)	2.03 (1.59-2.58) ***
Ovarian response& biomarkers				
Oocyte yield	(per 1 SD)	1.08(0.99-1.19)	1.07(0.91-1.25)	1.05 (0.95-1.15)
AMH	(per 1 SD)	1.12(1.03-1.22) *	0.95(0.82-1.11)	1.13 (1.03-1.23) **
BMI	(per 1 SD)	1.07(0.98-1.16)	1.01(0.87-1.18)	1.04 (0.95-1.13)
TG	(per 1 SD)	1.04(0.96-1.13)	1.00(0.87-1.16)	1.03 (0.95-1.13)
FSH	(per 1 SD)	0.98(0.90-1.06)	1.00(0.87-1.15)	0.97 (0.89-1.05)

All models were logistic regression models adjusted for all variables listed. Continuous variables were standardized (z-score transformation) prior to analysis; odds ratios therefore represent the change in odds per one standard deviation increase in the predictor. The standard deviations for the continuous variables in each cohort are provided in **Supplementary Table 14**. aOR, adjusted odds ratio; CI, confidence interval. *P < 0.05, **P < 0.01, ***P < 0.001.

## 4 Discussion

By integrating genetic causality inference with clinical mediation analysis, this study provides evidence for a causal role of triglycerides (TG) in lowering FSH levels and identifies a suppression pathway through which TG influences oocyte yield. Our principal finding establishes a causal direction from TG to FSH, and reveals that TG’s effect on oocyte yield is mediated through FSH in a manner that partially counteracts its direct negative impact.

Our MR analyses establish a causal direction from metabolic variation to ovarian dysfunction (17). The observed genetic associations (IVW β = -0.096, FDR = 5.28×10^-5^) helps to resolve a key uncertainty in the field. While observational studies have consistently correlated metabolic conditions with poor ovarian response (18–20), they could not preclude the possibility that these associations stemmed from confounding factors or reverse causation (21, 22). The association between metabolic status and lower gonadotropins has been previously described in fertile women; our study extends this evidence by using MR to establish causality and by quantifying the suppression effect in a DOR population (23, 24). Our MR findings, resilient in sensitivity analyses, strongly suggest that metabolic aberrations are an upstream contributor to the alterations in FSH.

The core mechanistic insight from this study is the discovery of a suppression effect in the TG-FSH-oocyte yield pathway. FSH is the primary hormonal driver of follicular recruitment and growth (25). In classic DOR, a decline in ovarian feedback leads to a compensatory rise in FSH (26). Our finding that TG causally lowers FSH presents a clinically important observation. We hypothesize that this reflects modulation of the hypothalamic-pituitary-gonadal (HPG) axis, potentially mediated by TG-associated metabolic disturbances such as leptin resistance or chronic inflammation, which can disrupt GnRH pulsatility or pituitary sensitivity (27, 28). However, the precise molecular mechanisms remain to be elucidated. The resultant state of “inappropriately low” FSH in the context of metabolic variation results in suboptimal for the already diminished follicle pool, further curtailing oocyte yield (29). The mechanisms are likely multifactorial, with potential contributors including estrogen negative feedback, leptin resistance, chronic inflammation, and hyperinsulinemia. Our data do not distinguish among these mechanisms. The precise molecular pathways remain to be elucidated (24). However, importantly, the suppression effect means that the FSH-lowering effect of TG partially offsets its direct negative impact—a nuance that has not been previously quantified in DOR patients.

These findings carry nuanced clinical implications. Establishing TG as an independent predictor of oocyte yield suggests that routine lipid screening could provide additional information in the diagnostic workup for women with DOR. The suppression effect further suggests that the relationship between metabolic status and ovarian response is more complex than previously appreciated. This identifies a potential therapeutic opportunity; interventions aimed at lowering TG, through lifestyle or pharmacological means, could be tested as a strategy to improve ovarian response (30, 31). However, our data also provide crucial context: the lack of an independent association between TG and ultimate live birth rates underscores that metabolic factors primarily impact the “quantity” of follicular recruitment, whereas the “quality” of oocytes and embryos—governed predominantly by age and intrinsic ovarian reserve—remains the critical determinant of cumulative live birth success (32, 33). Therefore, the clinical relevance of TG should be considered as one of many factors in a multifactorial assessment, rather than a dominant determinant.

Several limitations should be acknowledged. Our clinical cohort was retrospective from a single center with metabolic parameters measured at a single timepoint, and selection bias may arise from conditioning on DOR status (defined by AMH, AFC, or FSH), as these variables are also central to our mediation analysis (**Supplementary Figure 1**); while we cannot formally quantify this bias, the consistency of our findings across MR and multiple sensitivity analyses suggests it is unlikely to fully explain our results. Additionally, the exposure and outcome GWAS were not fully independent (both included UK Biobank participants), though the high F-statistics (>10) mitigate concerns about weak instrument bias, and the reverse MR analyses were underpowered due to the limited number of genetic instruments available for AMH and FSH, meaning that the absence of evidence for reverse causation should not be interpreted as evidence of absence. The suppression effect, while statistically robust, should be interpreted with caution given the modest effect sizes, and the pregnancy and live birth rates reported in this study reflect outcomes in the selected fresh-transfer subgroup and may not be generalizable to all DOR patients. Future prospective, multi-center studies with repeated metabolic assessments and intervention trials are warranted to confirm our findings, and deeper mechanistic investigations will be essential to unravel the precise molecular links.

## Data Availability

The original contributions presented in the study are included in the article/**Supplementary Material**. Further inquiries can be directed to the corresponding author.
